# The effect of habitat health and environmental change on cultural diversity and richness in animals

**DOI:** 10.1098/rstb.2024.0141

**Published:** 2025-05-01

**Authors:** Sofia Bolcato, Lucy Aplin

**Affiliations:** ^1^ Division of Ecology and Evolution, Research School of Biology, Australian National University, Canberra, Australian Capital Territory 2601, Australia; ^2^ Institute of Evolutionary Biology and Environmental Studies, University of Zurich, 8057 Zurich, Switzerland; ^3^ Cognitive and Cultural Ecology Research Group, Max Planck Institute of Animal Behavior, 78315 Radolfzell, Germany

**Keywords:** animal culture, social learning, conservation, anthropogenic change

## Abstract

There is increasing evidence that habitat decline via fragmentation or species loss can lead to loss of cultural diversity, complexity or richness in non-human animals. For example, a reduction in local bird species richness leads lyrebirds to sing fewer complex songs, while great apes living in fragmented landscapes have smaller cultural repertoires. However, the link between animal culture and local ecology remains understudied, and the potentially complex interactions between ongoing ecological change and animal culture are poorly understood. Here, we review the current state of knowledge on how ecology influences animal culture, focusing on vocal communication and foraging behaviour. We identify key factors affecting cultural patterning, including direct effects (e.g. environmental variability) and indirect effects (e.g. connectivity). We then review the emerging evidence for the effects of environmental change on culture, identifying three major threatening processes: habitat fragmentation, habitat degradation and urbanization. Finally, we develop a predictive framework for the effect of these threatening processes on animal culture and highlight how the loss of cultural diversity and complexity can lead to fitness costs with conservation implications.

This article is part of the theme issue ‘Animal culture: conservation in a changing world’.

## 1. Introduction

When innovations arise in human societies, they can spread rapidly to form new cultures, which can be retained and transmitted across many generations [1]. This capacity is thought to be vitally important in our success as a species, leading to a tight interweaving between culture and human adaptation termed the ‘cultural niche’ [2]. Yet while this is an exceptional example, culture is not exclusive to humans. Research over the last three decades has dramatically extended the taxonomic reach and breadth of culture [3–8]. This research has also revealed that animal cultures are not static but can change over time in response to Darwinian-like processes such as drift and cultural selection [9,10]. There is even a small but increasing body of evidence for the evolutionary implications of culture in animals, as a driver of reproductive isolation (e.g. in passerine birds: [11]), via gene–culture co-evolution [12], and as a factor in cognitive evolution [13]. Finally, recent research has identified the importance of retaining culture for conservation outcomes in some species [14–18], highlighting how culture can be a source of locally adaptive behaviour.

Culture is often defined differently depending on the research field and context, leading to potential confusion. In this article, we follow the accepted convention in animal ecology, defining culture as ‘group-typical behavioural patterns shared by members of a community that rely on socially learnt and transmitted information’ [19, p. 4]. We further define a cultural trait as a skill or behaviour that is socially learnt and retained at the individual level, and persists in groups over time, for example, via cultural inheritance across generations [20,21]. According to these definitions, cultural traits are fundamentally an emergent property of the interaction between social networks, cognition and the environment, and their form and patterning is an emergent consequence of these interactions [21–23]. Changes in resource landscapes, or an effect of the environmental change on social systems or demographics, should therefore have profound consequences for the form, diversity and resilience of cultures.

Given this close link between ecology and cultural patterning, moving towards a predictive framework for the interaction between the environment and culture is vital to incorporating animal culture into conservation decision-making. The most comprehensive recent attempt to do this has come from the formulation of the *disturbance hypothesis* [24], which argues, in the case of orangutans, that human impacts act as a triple threat to social connectivity, group sizes and resource availability. This then leads to reduced opportunities for social learning and subsequent loss of culture in a process coined the *fragility of traditions*. Yet, human activities can drive a diverse range of ecologically important behavioural responses in non-human animals [25,26], including promoting innovation through the provision of new opportunities [22,27,28]. Additionally, species that have responded to human impact by increasing in population size or density may be experiencing increasing opportunities for social learning [29]. Altogether, this could result in some scenarios of anthropogenic change that could lead to a complete reversal of the predictions of the disturbance hypothesis.

Here, we aim to bring these different forms and effects of environmental change on culture into a holistic and predictive framework. First, we review the evidence that culture is adaptive and review evidence for cultural adaptations. Second, we review the current state of knowledge for how ecology influences the emergence of culture, the patterning of culture and cultural complexity. Third, we focus on three distinct anthropogenic processes of habitat fragmentation, habitat degradation and urbanization, and explore their effects on culture. We aim to provide a starting point for conservationists and managers to form predictions for how culture will change under different habitat change scenarios and to consider the potential consequences of this feedback between culture and the environment.

## 2. Culture as locally adaptive behaviour

### (a) When is culture locally adaptive?

Most commonly, methods to identify culture focus on identifying differences in behaviour between populations while excluding other sources of potential variation between these populations (e.g. via genetics or ecology [30–32]). While this necessarily separates the link between local ecology and the presence of culture, parallel to this, theoretical work has long made a convincing case that cultures should typically be locally adaptive [33,34]. Most notably, Galef [33] argued that to be retained in an individual’s repertoire, behaviour should generally be beneficial. Given the expression of behaviour is necessary for others to copy it [35], the utility of behaviour should therefore determine its retention in the population. More recent work has extended this to argue that animals will not only selectively retain behaviour but also refine behaviour through ongoing practice, with this individual-level reinforcement learning leading to the transmission of more efficient and/or effective versions [36]. Finally, a long history of theoretical and empirical work has demonstrated that animals will often express evolved social learning rules, optimizing when, what and who to learn from in order to receive the most beneficial information [37].

Supporting this argument, while some studies have successfully seeded maladaptive information into groups, very few studies have demonstrated its ongoing retention. For example, in a controlled captive experiment in guppies, *Poecilia reticulata*, suboptimal longer movement routes were seeded into shoals but only persisted for a few days before individuals began to switch to the shorter route [38]. Similarly, great tits (*Parus major*) will switch to a more efficient or higher-reward version of a socially learnt foraging behaviour through selective retention and expression of the higher-payoff or more efficient solution [39,40]. Interestingly, growing evidence suggests social turnover is vital to this process, either by neutralizing behavioural conservatism [39,41] or by introducing naive individuals that might be more prone to innovate [42], allowing individuals to be exposed to a greater range of variants [39–41]. In the most extreme example of social turnover, Warner [43] replaced an entire local population of blue-headed wrasse (*Thalassoma bifasciatum*) in two separate experiments. He found that after the first replacement, a new set of communal mating sites was established, uncorrelated to the stable multi-generational traditions of the original population [43]. Yet, when this second population was replaced after only one generation, these mating sites were chosen again, suggesting that breaking established traditions allowed populations to resample and select the most locally adaptive behaviour at that time point [44]. He proposed that while socially learnt behaviour is generally locally adaptive, it might suffer from a time-lag effect he termed ‘cultural inertia’, which can be overcome with sufficient (in this case catastrophic) levels of turnover. More recently, this was modelled by Chimento & Aplin [41], who identified that social turnover does indeed promote cultural evolution by increasing repertoires and individuals’ ability to assess options; however, if turnover rate or tempo is too high, it risks behavioural extinction, with this risk amplified if behaviours are difficult to acquire or hard to reinvent.

### (b) Evidence for cultural adaptations?

Despite the evidence that culture is usually locally adaptive, there are relatively few examples of cultural adaptations as described in humans [2], where culture has facilitated establishment in new environments or persistence in changed environments [2]. Most documented cases involve local innovations that appear to be beneficial for survival or reproduction in changed environments [45,46]. Yet to give convincing evidence for a cultural adaptation would further require linking these cultural traits to fitness. There are only two case studies that come close to this. One example comes from populations of black rats (*Rattus rattus*) in pine forests in Israel. These pine trees lack native predators such as squirrels. Instead, invasive rats have a diet consisting almost entirely of pine nuts, which presumably facilitates their persistence in this new habitat. Experiments demonstrate that individuals socially learn to process pinecones, with behaviour vertically transmitted from mother to offspring. While the origins of this behaviour remain unclear, it is unlikely to be a pre-existing trait, given that other populations feeding on cypress cones cannot process pinecones [47–49]. [47–49] Second, a subset of a population of Indo-Pacific bottlenose dolphins (*Tursiops aduncus*) in Shark Bay, Western Australia, uses sponges as tools to forage for benthic fish in sand. This behaviour is transmitted from mother to offspring [50], and extends foraging into a different niche (sandy deep-water channels; [51]). In a recent marine heatwave, researchers found that tool users had higher survival than non-tool users, linking a cultural trait directly with fitness [52].

Socially learnt behavioural responses to urban environments also provide potential examples of cultural adaptations [29,45]. Here, the most convincing case study comes from the global phenomena of cultural evolution of passerine song in response to urban noise [53–55]. In the clearest example, the frequency of the song of white-crowned sparrows (*Zonotrichia leucophyrs)* living in San Francisco has increased over 30 years. Here, biased cultural transmission has favoured variants that can be heard over traffic noise, with males preferentially copying songs that are not masked by traffic and producing their own songs at higher frequencies. [53–55]. While changes in song frequency could partly reflect ontogenetic responses to urban noise (e.g. the Lombard effect), the evidence supports cultural selection. Specifically, while noise exposure did not prevent species-typical song learning, males exposed to masking noise preferentially replicated higher-frequency songs from tutors, suggesting active cultural selection rather than environmental adjustment during song ontogeny [53]. In controlled acoustic settings, these males paid a fitness cost relative to control males, as higher frequencies are less attractive to females, yet presumably in the cities, they have an adaptive advantage through maximizing signal transmission [54].

## 3. Linking culture with the environment

### (a) How is culture shaped by ecology?

If culture is locally adaptive, it further holds that its emergence, persistence, form and expression should be responsive to local ecological conditions. First, at the broadest level, the evolution of the capacity for culture is thought to be linked to intermediate rates of environmental change, with within-generation predictability but slow between-generational change selecting for learning over fixed behavioural patterns and for social learning over individual learning [56–58]. Alternatively, predictable fluctuations within generations can also be selected for social learning [57]; for example, in social ungulates, fluctuating environments with seasonal availability of high-quality resources have been argued to favour the evolution of culturally transmitted migration routes [59].

The importance of fluctuating resources and periods of resource scarcity for promoting the emergence and expression of culture has been best studied for foraging behaviour. Here, the *necessity hypothesis* argues that cultural traits tend to represent more complex or costly ways to access resources and so are more likely to have a selective advantage over other behaviours when used to access vital foods during periods of resource scarcity. In support of this, foraging innovations are more frequent in winter and in non-migratory species [60], and have further been linked to harsh climatic conditions and food shortage [61]. The link between ecology and culture has been most explicitly suggested for tool use in primates, where chimpanzees, capuchins and macaques have all been argued to rely on socially learnt tool use to access ‘back up’ foods when other options are not available [62–65].

However, this remains debated, with other studies suggesting a more significant link between foraging cultures and ecological opportunity [66,67]. The *opportunity hypothesis* posits that the opportunity and time to invent and express cultural behaviours will ensure their retention in populations. Supporting this, a higher density of resources requiring tools, such as ants or nuts, predicts tool usage in chimpanzees, [67] and zoo animals that have ample food and free time are often more innovative [68]. It seems likely that both hypotheses are possible. That is, if foraging cultures are more behaviourally complex and harder to learn than other forms of foraging (such as tool use), then they will most likely be used to access high-reward foods or foods still available when others are not. In the case of the former scenario, then there would not be a clear link with resource scarcity; instead, there may be an observed link with opportunity. In the second scenario, a link with necessity is more obvious. However, in both scenarios, some level of opportunity to express the behaviour, once it is invented, is needed to successfully retain it in the population.

Comparing between species or populations, the risk and time cost involved in learning and expressing cultural traits has led some authors to further link culture to the ecological conditions of *predator* and *competitor release*. First, a reduction in predation risk will allow individuals to lower vigilance, freeing individuals to direct attention towards social learning. Furthermore, individuals may be able to venture into new areas, developing new learnt behaviours to exploit the available resources. For example, capuchin monkeys (*Cebus capucinus*) on Coiba Island in Panama forage on the ground and use rocks to crack open shells, crabs and seeds, behaviours that are possible because of reduced predation risk on islands ([65], see also [69]). Second, release from interspecific competition can leave open ecological niches that individuals and groups can learn to exploit. For example, it is suggested that New Caledonian crows (*Corvus moneduloides*) developed tool use for extracting wood-boring grubs owiing to the absence of woodpeckers or other specialized extractive foragers on New Caledonia [70,71]. In both cases, we would therefore expect islands, which are often predator- or competitor-released, to be hotspots for animal culture.

### (b) Ecological influences on cultural complexity, richness and diversity

*Cultural complexity*, here defined as multi-component single cultural traits (e.g. in passerine bird song: [72]), *cultural richness*, here defined as multiple co-occurring cultural traits (e.g. foraging behaviours in chimpanzees: [73]) and *cultural diversity*, here defined as variation in cultural traits within and between groups [74], can all be shaped by ecology in a multitude of ways. These can be summarized as falling into two broad categories: direct effects of environment on culture and indirect effects via the effects of environment on social systems. Here we discuss these in turn.

#### (i) Direct effects of habitat

Beyond driving the emergence of culture through creating conditions of opportunity and necessity, habitat can directly shape within-population cultural patterning in two main ways. First, increased habitat heterogeneity and/or variability over space and time may support greater variability in cultural traits and overall increased cultural richness. In chimpanzees, for example, distance from Pleistocene forest refugia and long- and short-term seasonality are all associated with increased environmental variability and are further correlated with increased richness in cultural repertoires [75]. It is speculated that individuals that dispersed from these refugia likely encountered more environmental variability, promoting opportunities to innovate and retain additional cultural behaviours that facilitated adaptation to novel environments. In contrast, individuals remaining in the refugia experienced a more static environment with fewer opportunities for diversification and possible loss of cultural richness owing to the lack of sustained selective pressures or stochasticity.

Additionally, there is a positive correlation between behavioural diversity and seasonal variation [75], which has been linked to the need to exploit new and unpredictable food sources. This correlation between habitat variability and culture could occur within patches, increasing group-level repertoires, or at the landscape scale, where variation between habitat types across patches will promote greater cultural diversity at the population level. For example, in New Caledonian crows, tool manufacture is shaped by local plant and prey communities at relatively small scales [76].

Second, habitat productivity and biodiversity may influence cultural complexity. Two examples of this come from birds where males mimic heterospecifics as part of their socially learnt songs. In a study on two lark species (*Galerida* spp.), heterospecific taxonomic richness increased lark song complexity [77]. Reflecting this, in Albert’s lyrebirds (*Menura alberti)*, males in smaller patches with lower heterospecific abundance mimic fewer species [78], although in this case, the authors were unable to disentangle whether song simplification was driven by reduced biodiversity or fewer available conspecific tutors. Importantly, cultures may be influenced by both current and historical habitat features. In the case of a close relative, the superb lyrebird (*Menura novaehollandiae*), introduced populations on the island of Tasmania continue to mimic mainland heterospecifics more than 60 years after translocation [79].

#### (ii) Indirect effects of habitat

In addition to the direct effect of environmental conditions on culture, the environment can also influence population dynamics and structure, which can indirectly shape cultural outcomes. Perhaps most clearly, larger populations will have more opportunities for rare innovation events and are more likely to retain cultural behaviours owing to an increased pool of tutors and learners [80]. A larger population-carrying capacity (e.g. via larger continuous habitat patches or higher habitat productivity) should therefore support a larger cultural repertoire [80]. Conversely, smaller population sizes will be associated with cultural bottlenecks and drift, affecting the size and complexity of cultural repertoires. This is best studied in avian vocalizations, where, for example, islands with smaller populations often exhibit simpler and less diverse songs than mainland populations [81–84]. For instance, a reduction in population size has been associated with a decline in song diversity among Dupont’s lark (*Chersophilus duponti*) [85]. Here, song diversity positively correlated with population size, with individuals from small and less productive populations showing a relatively smaller vocal repertoire [85].

Whiten & van Schaik [86] proposed an association between gregariousness and cultural repertoire, with larger cultural repertoires being associated with more gregarious groups or species. If so, habitat health could also indirectly affect cultural richness via the ability to support larger group sizes. For example, higher habitat productivity is associated with increased social tolerance and party size among orangutans at Suaq Balimbing, Indonesia. This site, characterized by low seasonality in fruit production and high annual fruit yields, supports high population densities and significant home range overlaps, fostering greater social connectivity among individuals. In this context, the ecological conditions of and near the natal home range have been associated with larger party size (proxy for learning opportunities) and increased tool use specialization [87]. Similarly, chimpanzees exhibit variations in social tolerance and party size in response to ecological conditions, for example, with female foraging in groups primarily when resources are abundant [88]. Variation in tool use among chimpanzee populations correlates with social tolerance, with more time spent foraging in parties linked to greater cultural richness [88].

Finally, there is now increasing evidence that partial social connectivity, as observed between sub-populations in patchy habitats, is important for promoting cultural complexity. This has been long theorized in human social networks [89–91], and is matched with evidence from agent-based modelling showing that partially connected social networks enable both diversification of cultural traits within groups and recombination of these variants between groups into complex cultures [74,92–94]. This was recently explored in a study on chimpanzees, which found that cumulative cultural traits, but not simple cultural traits, were associated with limited levels of population interconnectivity among 35 chimpanzee communities in Central and West Africa [95].

### (c) Summarizing the main effects

**Figure 1.**
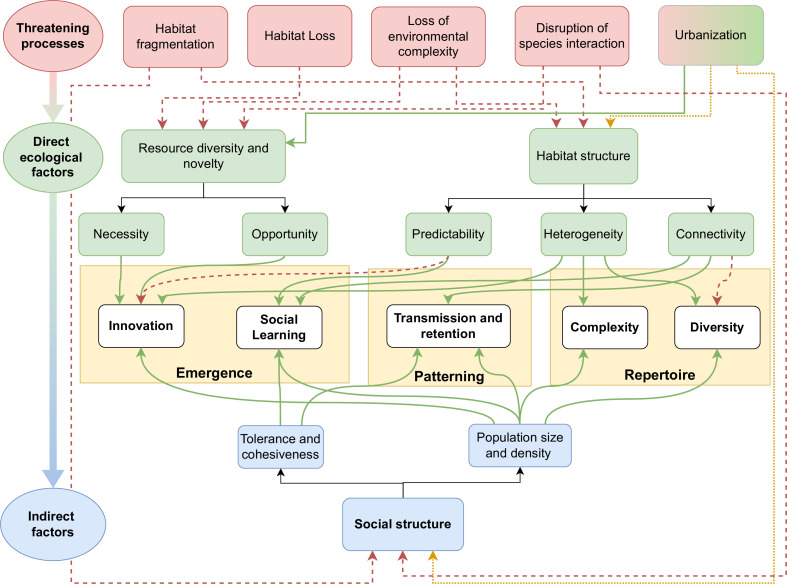
Predictive framework summarizing the relationship between threatening processes in red (top row), direct ecological factors in green (middle rows) and indirect social factors in blue (bottow rows) on the emergence, patterning of culture and cultural repertoires in yellow (boxes). Positive impacts are represented with green arrows (solid lines), negative relationships in red (dashed lines) and ambivalent relationships in yellow (dotted lines). Neutral relationships are shown with black solid lines.

The main effects of ecology and habitat topology on culture, as suggested by the current evidence, are expressed in figure 1 and can be summarized as follows. First, environments that experience moderate levels of variability over space and time, perhaps with short periods or areas of relative harshness, will tend to select for the emergence of culture and will tend to promote increased cultural richness within populations and diversity between populations. Second, habitats with greater biodiversity and productivity will provide more learning opportunities via direct effects (increased resource diversity) and indirect effects (larger group sizes giving more social learning opportunities). Coupled with this, larger habitat patches may also support larger overall population sizes, increasing the likelihood of innovation and retention of innovations, potentially leading to increased cultural richness. Finally, at the landscape scale, patchy habitats with intermediate levels of connectivity (e.g. through habitat corridors) will promote both cultural diversity and cultural complexity, as immigration between groups with different behavioural traditions leads to the potential for re-combinatory cultures and cultural accumulation.

## 4. Effects of anthropogenic environmental change on culture

Given the demonstrable effect of environmental variability, habitat productivity, patch size and between-patch connectivity on cultural outcomes, it follows that changes in these environmental characteristics will also tend to lead to cultural change and loss (figure 1). The negative effects of environmental change on culture were formalized for foraging traditions in orangutans (*Pongo* spp.) as the *disturbance hypothesis* [24]. This posited that declining populations under hunting or habitat degradation lead to lower innovation rates and reduced opportunities for social learning, and that habitat fragmentation reduces long-distance dispersal and therefore reduces social transmission [24]. However, more recent work has revealed that the impacts of environmental change on culture may be more multi-faceted in different taxa. Here, we concentrate on reviewing this evidence for three anthropogenic changes that are posing major global threats: habitat loss and fragmentation, habitat degradation and urbanization.

### (a) Habitat loss and fragmentation

Habitat loss is one of the most pervasive threats to biodiversity, driven primarily by deforestation, agriculture and urban development. The disruption of continuous habitats tends to result in habitat fragmentation, with patches reducing in size and with distances between suitable patches increasing [96]. For a given species, this will often result in decreasing population sizes and reduced movement and gene flow. As predicted under the disturbance hypothesis, this should lead to lower innovation rates and lower rates of social learning, reducing repertoire size in individuals and within-patch cultural richness [24]. However, if some level of connectivity between patches is maintained, it could conversely also lead to increased landscape-level cultural diversity and even potentially act to increase cultural complexity over the longer term. Supporting this, in one study on Dupont’s larks, Laiolo & Tella [85] found significantly fewer song types among individuals living in small patches of habitat. They further found that the introduction of anthropogenic barriers between patches has increased song similarity among neighbours and increased diversity between non-neighbouring individuals, leading to the emergence of micro-dialects within the same population [85,97]. These results are reflected in other studies in bird song that have highlighted a role for habitat loss and fragmentation in leading to changes in dialect diversity [98].

Alongside a reduction in within-patch cultural richness and an increase in between-patch diversity, the lower effective population sizes associated with habitat loss and fragmentation may also lead to increased cultural drift. This was recently noted in a 22-year study on social calls in two severely declining populations of yellow-naped amazons (*Amazona auropalliata*), where authors showed clear evidence for acoustic drift [99]. Interestingly, in this case, an expectation of acoustic divergence between fragmented populations was not supported, with results rather suggesting the opposite trend. This may have been due to an increase in long-distance movements, with birds roaming further in search of food or nest sites [99]. If so, it suggests that the predicted outcome will depend on an interaction between the degree of fragmentation, the dispersal abilities of the species of interest, the likelihood of reinvention of a cultural variant, and the degree to which habitat fragmentation affects other variables that influence learning or social connectivity.

### (b) Habitat degradation

In other cases of anthropogenic change, habitats may stay largely intact but gradually erode in quality and productivity via biotic and abiotic factors. Drivers of such changes include invasive species, overgrazing, selective logging, climate change and pollution. In many cases, habitat degradation will lead to a reduction in biodiversity and ecosystem complexity. This loss of biodiversity will likely result in a smaller carrying capacity, reducing population densities and leading to a similar effect to that described for habitat loss above. Additionally, habitat degradation may also directly change animal behaviour with detrimental effects for culture. For example, hunting and logging lead orangutans to become more solitary and secretive, decreasing social tolerance and disrupting social transmission of behaviour [24]. In addition to these effects, a decline in habitat productivity may also directly impact the potential range of behaviours that can be expressed, therefore reducing cultural richness.

In the most recent case study of these combined effects on foraging cultures, Kühl *et al.* [100] examined 31 behaviours across 144 chimpanzee communities and found that the probability of cultural behaviours occurring was reduced by 88% in areas with high human impact. While the authors could not disentangle specific drivers, they suggested that reduced population density, changes in plant phenology and nut availability, and lower carrying capacity due to habitat erosion all could have contributed to the loss of culture. This impact of habitat erosion on foraging culture has also been described in orangutans, where tool use is more common in areas with higher individual density and habitat productivity [101]. Finally, this dual threat can also be observed in vocal culture; in a recent study on Albert’s lyrebirds, impoverishment of the vocal repertoire was found in areas with lower proportions of suitable habitat, likely driven by both the biodiversity of heterospecifics to mimic and the density of conspecific tutors [76].

Importantly, unlike for habitat fragmentation, we would not expect population declines associated with habitat degradation to increase cultural diversity. Rather, a lower population density will more likely result in a breakdown of dialects and diversity [102]. For example, agricultural change in the United Kingdom has led to patterns of local extinction and recolonization in corn buntings (*Miliaria calandra*). When coupled with low social densities, these dynamics have resulted in a loss of previously observed vocal dialects [103]. Additionally, if cultural diversity is related to specializations of different resources [104], we might also expect homogenization of culture resulting from a reduced diversity in these resources.

Conversely, while habitat conversion can reduce habitat complexity and productivity, it can also introduce new human-derived resources such as introduced species, crops and human waste. Innovation and social learning of foraging behaviour in response to the introduction of these new resources have been reported in a large range of species, including black bears, *Ursus americanus* [105,106], sulphur-crested cockatoos, *Cacatua galerita* [45], bottlenose dolphins, *Tursiops* spp*.* [107], elephants, *Elephantidae* [108,109] and primates [108,110]. Culture is therefore a potentially important source of behavioural flexibility in response to these novel disturbances [29].

### (c) Urbanization

The introduction of novelty reaches its zenith in the process of urbanization. Urbanization, the movement of people to cities, and urban sprawl—the expansion of these urban areas—are replacing natural habitats with highly modified environments and represent an accelerating and globally significant challenge to biodiversity [111]. Over time, urbanization is associated with habitat loss, fragmentation, habitat homogenization and pollution, but with high availability of a subset of resources. Over time, resources in urban environments tend to be more predictable and less seasonal [27]*,* yet are vulnerable to abrupt change, for example from development. Finally, human areas have a high density of humans and their pets, which may contrastingly represent resource opportunities via direct feeding or waste for some species (e.g. garden birds), and a direct threat for others (e.g. rodents).

Nonetheless, despite these challenges, cities around the world have become refugia for species that can adapt to such novel environments [112]. Comparative analyses within and between species have shown that relative brain size and innovativeness are generally, although not always, positively correlated with urban colonization and persistence [61,113,114]. While still scarce, evidence is also beginning to emerge for the spread of the innovation and emergence of cultural traits around these new urban resources. For example, sulphur-crested cockatoos in Australia innovated how to open household bin lids to access food waste, with this spreading geographically to establish as a new cultural trait across southern Sydney ([45], see also Aplin *et al*. this issue [115]).

Given that there are relatively few studies of animal culture in urban environments, we have little empirical evidence for how animal culture will change in such environments. However, based on the observed forms of environmental change, we can make some predictions. First, urban environments tend to be patchy at the local scale, featuring a mosaic of gardens, buildings, parks and neighbourhood differences in planting. At this scale, resources can be abundant. This leads to a general tendency in urban-adapted animals for high social density, reduced movements and smaller home ranges [116,117]. Similarly to the effect of habitat fragmentation, we would expect this to lead to higher cultural diversity between patches. But in this case, this diversity should be coupled with a higher cultural richness within patches, driven by high social density leading to increased innovation and opportunities for social learning. However, that said, urban environments also tend to be homogeneous at the landscape level and tend to support relatively low species biodiversity [118]. This should lead to reduced cultural diversity at this scale, for example, in the degree of behavioural variation between cities.

Over time, cities tend to be less variable and less seasonal than comparable native habitats. Given that environmental variability is an important predictor of increased cultural richness within populations and diversity between populations, this would lead to the expectation that cultural richness and diversity would be reduced in urban areas. Additionally, while less climatically variable, resources in cities can experience abrupt changes, for example, when local governments act to remove weeds, poison pests or plant new street trees. For species that exhibit the rapid horizontal spread of information, this may promote higher rates of cultural evolution, enabling adaptive behavioural responses [22,29,119]. For instance, in the case of the bin-opening cultural behaviour in sulphur-crested cockatoos mentioned above, humans have responded by protecting bins, inducing cockatoos to learn how to defeat these measures in a potential innovation arms race [120]. However, for other species, where cultural traits are largely transmitted vertically from parent to offspring (e.g. as observed in great apes) [121,122], behaviour may not be able to keep track of such dramatic changes. Therefore, in urban environments, primary transmission modes for learning may be a major predictor of cultural outcomes [22] (table 1).

**Table 1.** Summary of predictions from §4 for the effects of habitat loss and fragmentation, habitat degradation and urbanization on the cultural metrics of cultural diversity, cultural complexity and cultural richness. Supporting evidence is provided, where existing.

environmental change	cultural metric	predicted impact	supporting evidence
habitat loss and fragmentation	cultural diversity	reduced within patches owing to smaller population size and increased drift potentially higher between patches owing to isolation	microdialects in Dupont's larks owing to fragmentation [85,97]
	cultural complexity	likely reduced owing to smaller effective population sizes and fewer opportunities for cultural recombination	variation in tool use among chimpanzees associated with social tolerance [86]
	cultural richness	reduced within patches owing to loss of tutors and learners may increase between patches if moderate connectivity allows recombination of cultural diversity	reduced cultural richness in orangutans in fragmented habitats [24]
habitat degradation	cultural diversity	reduced owing to declining population densities and resources homogenization	loss of vocal dialects in corn buntings owing to agricultural change [103]
	cultural complexity	reduced as degraded habitats may limit transmission of behaviours requiring high skill or specific resources	decrease in tool use by orangutans in areas with lower habitat productivity [101]
	cultural richness	reduced owing to fewer opportunities for innovation and reduced habitat productivity	loss of behavioural diversity in chimpanzee communities with high human disturbance [100]
urbanization	cultural diversity	higher between patches owing to local environmental heterogeneity lower at larger scale/across cities owing to landscape homogenization	cultural evolution of bird song [53–55]
	cultural complexity	higher within patches owing to higher density and innovation rates potentially reduced at broader scale in the absence of cultural recombination between patches	innovation arms race in sulphur-crested cockatoos' bin-opening behaviour [120]
	cultural richness	higher within patches owing to abundance of resources and opportunities for social learning potentially lower across the landscape owing to homogenization in resources	bin-opening behaviour in sulphur-crested cockatoos [45]

## 5. Conclusion

As explored in this article, there are complex interactions between culture, ecology and environmental change. This underscores the importance of considering cascading effects on cultural repertoires for conservation planning. Environmental changes induced by human activities have profound implications for the emergence, persistence and expression of animal cultural traits. Effects such as habitat fragmentation and degradation can have both direct effects on culture and indirect effects on sociality and behaviour, with these often combining to lead to an erosion of cultural diversity, richness and complexity.

Besides the intrinsic value of behavioural diversity, the loss of culture can have direct consequences on fitness for threatened species. Cultural traits often represent behavioural adaptations that have evolved in response to specific ecological challenges. These traits provide resilience to seasonal or unpredictable resources and enhance population adaptability, enabling individuals to exploit novel resources and adapt to new habitats. The erosion of cultural traits may therefore increase the risk of local extinction by reducing a population’s capacity to adapt to changing conditions. Our predictive framework illustrates the importance of maintaining diverse, complex and connected environments to support this cultural resilience in animal populations. However, it also highlights how the same effects may lead to anthropogenic environments becoming cultural hotspots for other species.

Overall, our paper highlights how, if conservation efforts aim to preserve the capacity for culture in species, this can be increasingly informed by a predictive framework, with this framework developed from multiple decades of theory and empirical evidence (figure 1). Shifting to this approach allows efforts to be targeted towards specific actions that protect social structures and environmental conditions that preserve cultural repertoires and foster cultural capacity.

## Data Availability

This article has no additional data.
